# Different patterns of P transposon and blood retrotransposon distribution in Harwich and Canton-S sub-strains do not affect the manifestation of Drosophila melanogaster intraspecific PM hybrid dysgenesis

**DOI:** 10.18699/vjgb-26-14

**Published:** 2026-03

**Authors:** L.P. Zakharenko, Y.Y. Ilinsky

**Affiliations:** Institute of Cytology and Genetics of the Siberian Branch of the Russian Academy of Sciences, Novosibirsk, Russia; Institute of Cytology and Genetics of the Siberian Branch of the Russian Academy of Sciences, Novosibirsk, Russia

**Keywords:** PM hybrid dysgenesis, Drosophila melanogaster, P-element, blood retrotransposon, PM гибридный дисгенез, Drosophila melanogaster, P-элемент, ретротранспозон blood

## Abstract

Intraspecific infertility, the nature of which is not always understood, occurs in many eukaryotes. Intraspecific PM hybrid dysgenesis (PM HD) in Drosophila melanogaster manifests in one cross direction as offspring infertility and other genetic disorders due to incompatibility between the maternal cytoplasm and the paternal genome. PM HD is believed to result from a massive transposition of the P-element when the maternal cytoplasm lacks a repressor to block it. In this work, we have investigated the distribution of the P transposon and blood retrotransposon in the reference PM HD strains (Canton-S and Harwich), which have been maintained in different laboratories for several decades. P-element distribution patterns vary among Harwich sub-strains, indicating that the P-element was translocated in these genomes. The rate of movement of the P-element, which was not induced by crosses, is comparable to the rate of movement of other DNA transposons. The distribution pattern of the low-active blood retrotransposon in Harwich sub-strains is more stable than that of the P-element, indicating genetic relatedness between sub-strains. Derivatives of the P-element detected in some Canton-S sub-strains possibly indicate genetic contamination. The significant difference in the blood transposable element distribution pattern in Canton-S sub-strains also indicates genetic heterogeneity among them. Despite the complex genealogy of the studied sub-strains, including cases of possible genetic contamination, and differences in P-element distribution, the ability to express PM HD symptoms is preserved in the studied sub-strains.

## Abstract (ru)

Внутривидовое бесплодие, природа которого не всегда понятна, встречается у многих эукариот. Внутривидовой PM гибридный дисгенез (РМ ГД) у Drosophila melanogaster проявляется в одном из направлений скрещивания в бесплодии потомства и некоторых других генетических нарушениях в результате несовместимости между материнской цитоплазмой и отцовским геномом. Считается, что PM ГД является результатом массового перемещения P-элемента, если материнская цитоплазма не имеет репрессора, блокирующего его перемещение. В данной работе мы исследовали распределение P-элемента в отводках референсных для PM ГД линиях (Canton-S и Harwich), которые долгое время содержались в разных лабораториях. Паттерны распределения P-элемента различались в разных отводках Harwich. Скорость перемещения P-элемента, не индуцированная скрещиваниями, сопоставима со скоростью перемещения других ДНК-транспозонов. Паттерн распределения малоактивного ретротранспозона blood в производных линии Harwich был более стабильным по сравнению с Р-элементом, что свидетельствует о генетическом родстве между отводками. В некоторых отводках линии Canton-S были обнаружены следы P-элемента, что может указывать на генетическое загрязнение. Значительная разница в паттерне распределения транспозона blood в отводках линии Canton-S также говорит о генетической гетерогенности отводков. Несмотря на сложную генеалогию отводков исследованных линий, включающую случаи возможного генетического загрязнения, и различия в распределении P-элементов, способность проявлять симптомы PM ГД у исследованных отводков сохраняется. Мы показали, что все изученные производные Canton-S имеют сильный M-цитотип, а все отводки Harwich – индуцирующий P-цитотип

## Introduction

Progeny of some Drosophila melanogaster suffer from the hybrid dysgenesis (HD) phenomenon that occurs in one cross direction (Kidwell et al., 1977) and results in female sterility, male recombination, high mutation rate and other genetic disorders (Bingham et al., 1982). It is commonly considered that HD is caused by massive P, hobo or I element transpositions in PM, HE or IR HD, respectively (Bingham et al., 1982; Bucheton et al., 1984; Blackman et al., 1987).

It is a commonly held opinion that P-elements appeared in the D. melanogaster genome by horizontal transfer in the middle of the last century. In American populations, P-elements were first identified in D. melanogaster obtained from nature in 1938, from Russian populations, in 1966, and from French populations, in 1967 (Anxolabehere et al., 1988). How-ever, all D. melanogaster strains collected on different continents from 1969 contain P-elements in their genomes (Anxolabehere et al., 1988). Thus, the distribution of the P-elements throughout the world was extremely rapid despite possible harm.

PM is the most common variant of HD, manifesting with all symptoms listed above. It was postulated to occur in crosses between females with the M cytotype, which lack P-elements in their genome, and males that harbor a functional P-element (Bingham et al., 1982). However, it was later found that many M cytotype strains contained a complete P-element (Bingham et al., 1982), and the presence of a P-element in the genome did not guarantee the induction of HD (Itoh et al., 1999, 2001, 2007). For this reason, the nature of PM HD is studied mainly on the reference Canton-S (Maternal cytotype) and Harwich (Paternal cytotype) strains. Crossing these strains results in HD phenotypes with 100 % probability. Not every strain with a full-length P-element in its genome induces HD, and none of them consistently exhibits HD as strongly as the Harwich strain. This fact alone casts doubt on the hypothesis of the P-element’s involvement in HD induction. A correlation was found between the strains’ ability to induce PM HD and the presence of a highly truncated P-element variant named “Har-P” in the Harwich genome, which is the most frequent de novo insertion. However, genomic location may also influence host tolerance of Har-Ps, as a significant rescue of viable pupae was observed in crosses with Har-Ps located only on Chromosome 3, which has six P-elements, while no pupae survived with Har-Ps located on Chromosome 2, which has nine P-elements (Srivastav et al., 2019). Thus, the reason for the uniqueness of the reference P strains remains not fully understood. According to a widely accepted hypothesis, the P-element mobilization is repressed in P cytotype strains by small RNA (Brennecke et al., 2008; Khurana et al., 2011).

The Canton-S strain has been widely used among genetic scientists since 1925. However, P-elements have been identified in some Canton-S strains (Ignatenko et al., 2015), possibly as a result of genetic contamination during cultivation in laboratory. “A golden rule of Drosophila genetics is never trust a stock label, no matter how reputable the source from which it was obtained ” (Ashburner, 1989).

Here, we analyze the pattern of P transposon and blood retrotransposon distribution in Canton-S and Harwich substrains from different laboratories and their ability to produce dysgenic progeny to describe accumulated genetic differences. We selected blood retrotransposon, as its distribution pattern remained stable for two decades in spite of the presence of a complete blood copy in the isogenic reference y; cn bw sp genome (Ignatenko et al., 2015). According to our data, the rate of movement of the P-element in Harwich sub-strains without dysgenic crosses is comparable to the rate of movement of other DNA transposons. All Canton-S derivatives have a strong reactive M cytotype, and all Harwich sub-strains have an inducer P cytotype.

## Methods

Strains of Drosophila melanogaster. Five Canton-S (CS) and three Harwich (H) sub-strains from different laboratories were used for the study (see the Table). The H2 sub-strain has a visible, spontaneously occurring mutation in the sepia gene (66 D5), and the H3 sub-strain has a mutation in the white gene (3 B6). There is no information in the literature about the artificial origin of these mutations. The LK-P (1A) strain containing one complete P-element per genome (from S. Ronsseray) was used to obtain a DNA probe of a full-sized P-element. Reference strain y; cn bw sp (No. 2057, Bloomington Drosophila collection) with a completely sequenced genome was used to obtain a blood probe (see the Table). The Harwich strain was taken from nature (Harwich, Massachusetts) in 1967 by M.L. Tracey (Kidwell et al., 1977). According to S. Ronsseray, the Harwich strain descended from two females (Ronsseray et al., 1984). Bloomington Drosophila Stock Center received the Harwich strain (stock number 4264) from M. Kidwell in 1997.

**Table 1.**
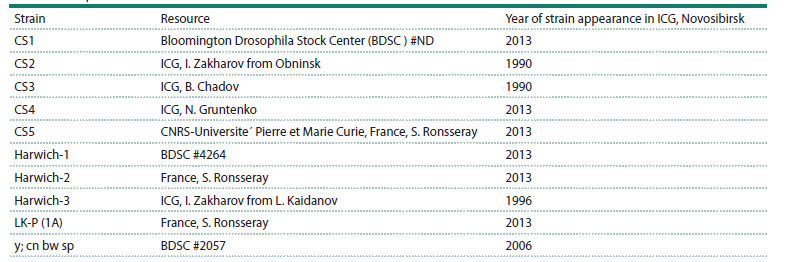
Strains description

Hybrid dysgenesis assays. To test HD sensitivity, substrains were crossed to CS2 or H3, a cross between which results in high penetrance of HD (Ignatenko et al., 2015). Progeny from crosses between H3 males and females of each Canton-S sub-strain, as well as CS2 female and males of each Harwich sub-strains, were cultivated at 29 °C from the egg stage to adulthood. As a marker of PM HD, ovary morphology was analyzed in 50 females aged 4–5 days (Kidwell, Novy, 1979).

Fluorescence in situ hybridization (FISH). The P-element probe was obtained by PCR with a primer complementary to terminal inverted repeats 5′-TGATGAAATAACATAAGGTG GTCCCGTCG-3′ (Takasu-Ishikawa et al., 1992; Lapie et al., 1993), and the blood probe, with primers 5′-CAGTGGCATAC GCTTCAAGA-3′ and 5′-GGTTCGCGAAATACCAGTGT-3′ (Ignatenko et al., 2015). The probes were labeled by nicktranslation with digoxygenin-dUTP for the P-element, and with biotin-dUTP for blood. FISH analysis was performed for 3–5 larvae for each sub-strain as in Ignatenko et al. (2015). Squashed preparations of polytene chromosomes from the salivary glands of third-instar larvae were made according to standard procedures (Biémont et al., 1994). The hybridization sites were analyzed using an Axio Imager M1 (Carl Zeiss) microscope in the Shared Facility Center for Microscopic Analysis of Biological Objects SB RAS, Novosibirsk.

## Results

To test sub-strains for PM HD, they were crossed to CS2 or H3, a cross between which results in high penetrance of HD (Ignatenko et al., 2015). Females of the CS2 sub-strain were crossed to H1, H2 or H3 males to check Harwich sub-strains induction ability. Females of five Canton-S sub-strains were crossed to H3 males to check the ability of Canton-S substrains to respond to induction. Indeed, abnormal gonad morphology was observed in progeny of all dysgenic crosses with 100 % penetrance, indicating a strong P cytotype for Harwich and M cytotype for Canton-S sub-strains (Table S1)1. Opposite crosses (female Harwich to Canton-S males) produced normal progeny (Table S1).

Supplementary Materials are available in the online version of the paper: https://vavilovj-icg.ru/download/pict-2026-30/appx2.pdf


**Fig. 1.**
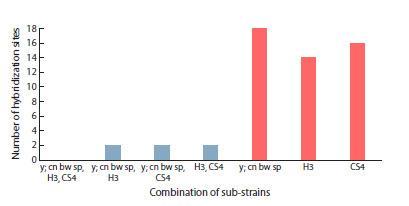
Distribution of common and unique blood hybridization sites in unrelated strains

**Blood element pattern in Harwich and Canton-S sub-strains** The pattern of the blood retrotransposon was studied to determine the level of similarity among the sub-strains. The reason for choosing the blood TE was its low-level mobility observed in the isogenic reference strain y; cn bw sp. The localization of the blood retrotransposon in the y; cn bw sp strain has not changed significantly over two decades (1992–2013) (Ignatenko et al., 2015). As expected, unrelated strains (H3, CS4 and y; cn bw sp) display mostly unique blood hybridization sites (89 %), and no hybridization sites were found to be common to all three strains. Pairwise matching sites were rare (Fig. 1, Tables S2, S4).

**Fig. 2.**
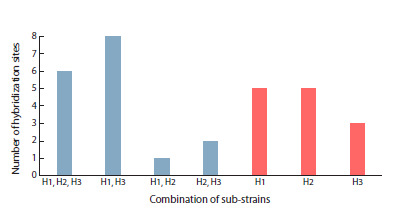
Distribution of common and unique blood hybridization sites in Harwich sub-strains

Thirty two blood hybridization sites were found in the genomes of Harwich sub-strains: 6 sites common for all three sub-strains, 15 unique sites, and 11 sites common for different pairs (Fig. 2). Thus, 20 % of blood hybridization sites are common to all three sub-strains (H1, H2, H3), and 47 % are unique. The H1 and H3 sub-strains displayed the greatest similarity, harboring 56 % (14 out of 25) common hybridization sites. It can thus be assumed that of the three investigated Harwich substrains, at least H1 and H3 sub-strains have common origin.

**Fig. 3.**
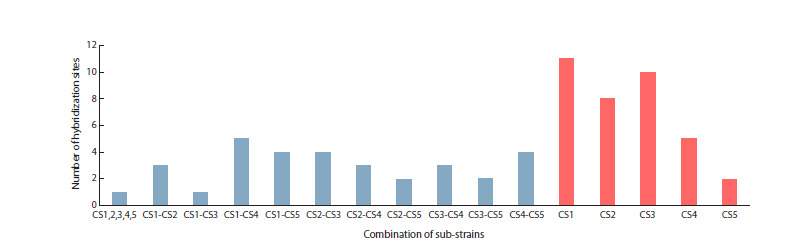
Distribution of common and unique blood hybridization sites in Canton-S sub-strains.

Strikingly, the blood distribution pattern shows extreme variability among Canton-S sub-strains. Of the total 60 hybridization sites, most are unique (60 %). One site is shared by all five sub-strains, two sites are shared by three sub-strains, and pairwise coincidences range from one to six common sites between different pairs (Fig. 3). Consequently, genetic contamination of at least some sub-strains cannot be excluded. Additional evidence for genetic contamination comes from the presence of traces of the P-element in some of these Canton-S sub-strains (Ignatenko et al., 2015) despite the fact that classically Canton-S serves as the gold standard for being P-elementnegative. It is interesting that traces of the P-element (two sites per genome) were found only on the third chromosome and were localized in different regions of the CS1, CS2, CS4 genomes (Ignatenko et al., 2015). Notably, P-element appeared in the D. melanogaster genome later than the Canton-S strain was isolated from nature. Rahman with coauthors (2015) also reported introgression of P-element-containing lab strains into certain stocks of Oregon R, which should be free of P-elements originally (Robertson, Engels, 1989).

**P-element pattern in Harwich sub-strains**

**Fig. 4.**
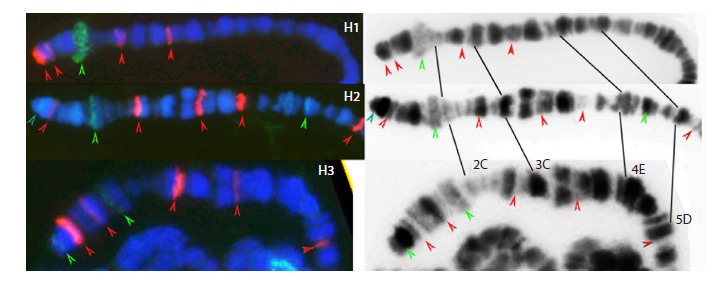
Localization of P-element (red) and blood TE (green) hybridization sites on the end of the X chromosomes of Harwich sub-strains from different laboratories. DAPI (blue).

**Fig. 5.**
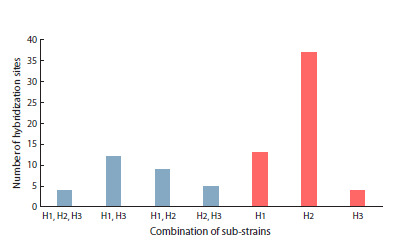
Distribution of common and unique P-element hybridization sites in Harwich sub-strains

We identified 85 P-element hybridization sites in Harwich sub-strains, of which only 5 % were common for all three sub-strains (Fig. 4, Table S3, Figs S1–S3). The percentage of unique P-element hybridization sites is 33 % for H1, 69 % for H2 and 28 % for H3 (Fig. 5). This result shows much higher variability in P-element distribution compared to blood distribution among the Harwich sub-strains. Moreover, common P-element hybridization sites, for example in 3F regions, show weak signal in the H3 sub-strain, suggesting a possible difference in fine structure of P-elements in different sub-strains (Fig. 4). Although FISH is a semi-quantitative method, the use of a full-length copy of the P-element as a probe allows interpreting the intensity of the signal as a reflection of the size of the sequence with which the probe hybridizes on the chromosome. Thus, the difference between sub-strains in the distribution of hybridization sites may be greater than we assume.

The H2 sub-strain harbors the largest number of unique P-element sites (44 %), which is consistent with blood distribution in this sub-strain also being the most distinct from the other two Harwich sub-strains in pairwise comparisons. In turn, the H1 and H3 sub-strains, which show the greatest similarity in blood distribution, shared 35 % (16 out of 47) of P-element hybridization sites. Thus, although the Harwich sub-strains are more heterogeneous in P-element distribution compared to blood distribution, a closer relationship between the H1 and H3 sub-strains is suggested by distribution patterns of both retrotransposons. Besides, the H2 and H3 sub-strains have visible genetic markers that help to monitor the purity of the strains. H3 has a mutation in the white gene, H2, in the sepia gene. The origin of the sepia and white mutations has not been precisely determined. The H3 strain was obtained from L. Kaidanov in 1996. Similarities in P-element distributions at the end of the X chromosomes of different Harwich derivatives support the spontaneous occurrence of the white mutation at least (Fig. 4).

Three Canton-S sub-strains (CS1, CS2, CS4) contain P-element derivatives as a result of possible genetic contamination. Given that derivatives of P-element were found only on the third chromosome, these sub-strains may share a common origin and may have been distributed between laboratories after P-element contamination. In terms of blood distribution, the CS1 and CS4 sub-strains show the highest degree of similarity among all pairwise comparisons. CS3 and CS5 have no traces of the P-element in the genome; this circumstance makes them similar.

## Discussion

We found that all Canton-S sub-strains have strong M cytotypes and all Harwich sub-strains exhibit a strong P cytotype independent of possible genetic contamination and a different pattern of P-element distribution. A comparison of the Harwich sub-strains indicates that the P-element can change its position without crossing with M strains. This is similar to the genomic instability reported for I-elements, which also occurs in the absence of dysgenic crosses (Moschetti et al., 2010). The symptoms of IR (Inducer-Reactive) HD differ from those of PM HD: dysgenic females lay eggs, but the offspring die at the embryonic stage. In both cases, the symptoms are consistently reproduced with the same physiological manifestations typical of a given HD type and specific reference strains. All other things being equal, only a limited number of strains can induce HD in both cases

The Harwich strain was separated from nature (Harwich, Massachusetts) in 1967 by M.L. Tracey (Kidwell et al., 1977). The peak of activity in studying the PM HD phenomenon and the spread of the strain across laboratories occurred in the 90s of the last century (Anxolabehere et al., 1984, 1988; Wang et al., 1993; Simmons et al., 1996; Ronsseray et al., 1998). During this time, at least 300 generations of Drosophila have changed. The H1 and H3 sub-strains differ in 11 out of 25 common blood hybridization sites, and in 31 out of 47 P-element sites. In our case, the rate of transposition can be estimated as at least as 15×10–4 (11/25/300) per site per genome per generation and 22×10–4 (31/47/300) for blood and for P-element, respectively.

According to the FISH analyses, the rate of TE transpositions in natural populations varies by several orders of magnitude, ranging from 10−5 per copy per generation to 10−2 per copy per generation (Pasyukova et al., 1998; Maside et al., 2000; Díaz-González et al., 2011). Sequencing analysis of parents and offspring from 18 families of full-sib D. melanogaster estimated the range of TE insertion rate from 10−3 per copy per generation to 10−5 per copy per generation (Wang et al., 2023). By comparing the parents and offspring in each family, authors identified 89 new TE insertions across the 89 samples, making it only one insertion per genome per generation on average (Wang et al., 2023). The rate of movement of the P-element in our experiment is in the range typical for the movement of other TEs.

Whole genome sequencing was used to estimate the rate of P-element movement in dysgenic ovaries and germ cells (Moon et al., 2018; Jansen et al., 2024).

Contrary to predictions based on the insertional mutagenesis model of hybrid dysgenesis, single-cell whole-genome sequencing analysis of DNA from dysgenic and non-dysgenic embryos at late embryonic stages (before dysgenic germ cell death) shows that dysgenic and non-dysgenic germ cells acquire unexpectedly similar, low numbers of new heterozygous P-element insertions (Jansen et al., 2024). The authors found double-strand breaks in generative cells and proposed that transposase excises the P-element, but does not integrate it into the genome (Jansen et al., 2024). To confirm their assumption, the authors simulate the excision of the P-element by using CRISPR Cas9. However, the gaps with blunt ends, induced by CRISPR Cas9, are usually completed by a non-homologous path (Quétier, 2016), whereas transposase recognizes the inverted terminal ends of the P-element and cuts them with the formation of sticky ends, which are successfully ligated or completed by reparation enzymes using the homolog. The excision of the P-element can occur precisely, without loss of gene functionality (Weinert et al., 2005). Double-strand breaks in dysgenic germline cells can be induced not only by P-element translocation.

Moon with coauthors investigated the rate of P-element movement in the ovaries of dysgenic females raised at 18 °C, when symptoms of hybrid dysgenesis do not appear and the ovaries have normal morphology (Moon et al., 2018). Nevertheless, the authors found 527 new P-element insertion sites in dysgenic ovaries. However, only a single new P-element insertion was found in F1 and F2 dysgenic progeny (Eggleston et al., 1988). It means that cells with a huge number of new P-elements should be eliminated, but the ovaries of dysgenic females raised at 18 °C, paradoxically, have normal morphology. Thus, disputable results were obtained about the mass movement of P-elements during PM HD.

It should be noted that Moon with coauthors do not see the movement of other TE families in the dysgenic cross direction (Moon et al., 2018).

The Harwich genome contains 132 P-elements according to Moon, and 80 (half of which are heterozygous) according to Jansen with coauthors (Moon et al., 2018; Jansen et al., 2024). On the one hand, the annotation of sequenced genomes with repeats is still imperfect and the difference can be explained by a reading error. For example, Moon with coauthors (2018) admit that 34 new insertions per genome may be false positives. On the other hand, it is possible that the issue is not only in the accuracy of the sequencing method or the choice of tissue for sequencing but also that the Harwich sub-strains, although having the same origin, were cultivated in different laboratories for a long time. Jansen with coauthors used the Harwich strain from BDSC. According to Ronsseray, this strain descended from two females since 1967 (Ronsseray et al., 1984). The initial polymorphism of the strain may affect the heterogeneity of the sub-strains. Moon (Moon et al., 2018) received the Harwich strain from Khurana et al. (2011), Khurana (Khurana et al., 2011) received the Harwich strain from Ronsseray. Thus, sub-strains originate from the same source, but have been cultured independently for a long time.

According to our data, Harwich sub-strains from different laboratories also differ in the number and localization of P-element hybridization sites. The sub-strain obtained from Ronsseray has more P-element hybridization sites than the substrains from BDSC. This is less than that revealed by sequencing, because the sensitivity of the FISH method depends on the specific activity of the labeled probe. In addition, weak signals may be missed during FISH analysis. Nevertheless, according to our data, relationship between the Harwich sub-strains is undeniable, since the percentage of common hybridization sites between the Harwich sub-strains is higher than between obviously unrelated strains.

PM HD may result not only from possible massive P-element movement, but also from an incompatibility of temperature- dependent metabolic processes of sensitive to PM HD strains, as dysgenic flies, when reared at low temperatures (20 °C), do not display dysgenic symptoms (Engels, Preston, 1979; Dorogova et al., 2017). Additionally, there is a hormonal difference between dysgenic and non-dysgenic flies (Zakharenko et al., 2014). The reference PM HD Harwich and Canton-S strains also differ in a number of physiological characteristics: life expectancy, fertility, locomotor activity, development rate (Zakharenko et al., 2024). The asymmetry in the expression of the dysgenic traits, characteristic of interspecific hybrids, suggests a significant genetic distance between the reference PM HD strains not only in P-element absence/presence

## Conclusion

The relationship between Harwich sub-strains is undeniable despite the difference in P-element distribution pattern. The rate of movement of the P-element in Harwich sub-strains without induction by crossing is in the range typical for the movement of other TEs. All Canton-S sub-strains have strong M cytotypes and all Harwich sub-strains exhibit a strong P cytotype independently of possible genetic contamination and a different pattern of P-element distribution

## Conflict of interest

The authors declare no conflict of interest.
